# Ketosis as a Treatment for Severe Mental Illness: A Critical Review of Preclinical and Clinical Data

**DOI:** 10.1007/s40501-026-00394-z

**Published:** 2026-08-17

**Authors:** Ifeoluwa Oluwasegun Oduguwa, Daniel J. Smith, Arish Mudra Rakshasa-Loots

**Affiliations:** Division of Psychiatry, Institute of Neuroscience & Cardiovascular Research, University of Edinburgh, Edinburgh, UK

**Keywords:** Ketogenic, Ketosis, Severe mental illness, Bipolar, Depression, Schizophrenia

## Abstract

**Purpose:**

Ketogenic interventions, including ketogenic diets, are increasingly being explored as potential treatments for severe mental illnesses. Ketosis – the metabolic state characterised by high circulating levels of ketone bodies – is thought to be central to the beneficial effects of these diets. Hence, it is plausible that other interventions that induce ketosis may similarly improve mental health outcomes. Here, we critically appraise the evidence surrounding the use of ketosis-inducing interventions for the treatment of severe mental illness.

**Recent Findings:**

Several uncontrolled trials suggest beneficial mental and metabolic health effects of ketogenic diets in depression, bipolar disorder and schizophrenia, but are limited by confounders and expectation bias. The only reported randomised controlled clinical trial so far suggested a small, greater between-group difference in antidepressive effect of a modified ketogenic diet over a phytochemical control diet in treatment-resistant depression, that did not reach statistical significance. Additionally, ketone levels did not correlate with psychiatric outcomes. Preclinical studies suggest a beneficial effect of ketogenic diets on psychiatric symptom correlates but largely did not explore ketone-outcome correlations. Exogenous ketone and oral medium-chain triglyceride supplements induce nutritional ketosis, however, they have not been tested in patients with severe mental illness. Fasting and time-restricted eating produce ketosis but it is unclear if this explains their reported mental health and metabolic effects.

**Summary:**

Large, randomised controlled clinical trials are needed to examine and compare the effects of different ketogenic interventions in severe mental illness. Trials should be designed to isolate the role of ketosis, investigate the mechanistic basis of beneficial effects observed, assess dose-response relationships, examine treatment adherence in routine clinical settings, ascertain the duration of treatment effects following discontinuation, and clarify the impact of ketosis-inducing interventions on the need for psychiatric medications and standard care.

**Supplementary Information:**

The online version contains supplementary material available at 10.1007/s40501-026-00394-z.

## Introduction

The emerging field of metabolic psychiatry aims to characterise the relationships between metabolism and mental health, to inform new or improved interventions for psychiatric conditions [1]. Ketogenic interventions, including ketogenic diets, are increasingly being explored as potential treatments for severe mental illness. These diets have been implemented clinically since the 1920s for treatment-resistant epilepsy [2] and cause the brain to use ketone bodies rather than glucose as its predominant energy source. Their use in psychiatric conditions is supported by shared neurobiology with epilepsy including neurotransmitter imbalance, mitochondrial dysfunction, chronic inflammation, oxidative stress, and abnormal metabolism [3–5]. Early investigations of ketogenic diets have shown promise for treating severe mental illness in preclinical and clinical studies.

Ketosis, the metabolic state characterised by high circulating levels of ketone bodies, is thought to be central to the beneficial effects of these diets. Ketone bodies administered in preclinical models and clinical studies of neurodegenerative conditions such as Alzheimer’s disease [6] and Huntington’s disease [7], and metabolic diseases such as obesity [8] produce similar therapeutic brain health and metabolic effects to those associated with ketogenic diets [9, 10]. Hence, it is plausible that ketosis itself might be the primary means by which ketogenic diets exert their therapeutic effects in severe mental illness, and that other ketosis-inducing interventions may have comparable efficacy to ketogenic diets. This narrative review therefore aims to critically discuss the evidence surrounding the use of ketogenic diets and other ketosis-inducing interventions for the treating severe mental illness.

## Literature Search

We conducted a literature search of PubMed from database inception to April 15, 2026, using the following terms: (ketosis OR ketogenic OR exogenous ketone OR medium-chain triglyceride OR time-restricted eating) AND (severe mental illness OR SMI OR bipolar OR schizophrenia OR schizoaffective disorder OR psychosis OR psychotic disorder OR major depression). Search results were independently screened by authors IOO and AMRL. Disagreements in screening decisions were resolved by discussion and consensus. Studies were included if they met one or more of the following criteria:

- preclinical studies evaluating ketosis-inducing interventions in validated animal models of schizophrenia, bipolar disorder, or depression,
- clinical trials reporting ketosis-inducing interventions in populations with schizophrenia, bipolar disorder, or depression.
- Preclinical and clinical studies evaluating the effects of fasting and time-restricted eating on mental health.

Studies were excluded if they were observational in design or published in languages other than English. This meant that we excluded case reports and case series [11–14] which typically report outcomes in one or a few individuals without a predefined systematic protocol in favour of studies offering a more systematic assessment of intervention effects. Additional eligible publications were identified using manual citation searching in included studies. In total, our systematic search yielded 540 articles, of which 20 were eligible studies included in this review.

## Ketogenic Interventions and their Application to Severe Mental Illness

Several dietary and nutritional interventions can induce ketosis, differing in their practical requirements, acceptability, and strength of evidence for efficacy in severe mental illness. For the purposes of this review, we consider four categories: ketogenic diets, which induce ketosis through dietary carbohydrate restriction; exogenous ketone supplements, which deliver ketone bodies directly without dietary restriction; oral medium-chain triglyceride compounds, which promote endogenous ketone production through rapid fatty acid oxidation; and fasting and time-restricted eating, which induce ketosis through periods of caloric abstinence or restriction.

### Ketogenic Diets

The classic ketogenic diet was first implemented in 1921 for treating drug-resistant epilepsy [15]. It derives 80–90% of its caloric content from fat, 6–15% from protein, and 5–10% from carbohydrates [16]. It contains 3 or 4 times as much fat as carbohydrates and protein combined and does not involve fluid restriction. Long-chain triglycerides are its primary fat source, and it is introduced with small, high-fat meals following a period of fasting. Although this diet effectively reduced seizure frequency, the strict dietary restrictions involved limited its acceptability, leading to the development of less stringent ketogenic diet variants such as the medium-chain triglyceride ketogenic diet, the modified Atkins diet, and the low glycaemic index treatment.

The medium-chain triglyceride ketogenic diet was introduced in the 1970s [17] and derives 60% of its calories from medium-chain triglyceride oils (which contain 6–12 carbon atoms) rather than long-chain triglycerides [18]. Since the former are more ketogenic, the medium chain triglyceride ketogenic diet allows a lower ratio of fat to carbohydrates and protein (1.2:1), improving acceptability and permitting more varied food choices than the classic ketogenic diet [19]. It also produces similar levels of ketosis with comparable efficacy and side-effect profile to the classic ketogenic diet in the treatment of intractable epilepsy [18–21]. It is a whole-diet approach distinct from oral medium-chain triglyceride supplementation combined with an unrestricted diet, which is discussed separately in this review.

Other variants of ketogenic diets include the modified Atkins diet and the low glycaemic index treatment. The former encourages liberal fat intake, avoids fluid or caloric restriction, and limits carbohydrate intake to 10 g/day during the first month of its implementation [22], with subsequent gradual increments in carbohydrate (up to 20 g/day or 19% of total energy) and fruit and vegetables over the treatment period [16, 22]. It differs from the Atkins diet in that carbohydrate intake, although more generous than in the classic and medium-chain triglyceride ketogenic diets, remains limited throughout the duration of its implementation [23].

The low glycaemic index treatment derives 60% of its calories from fat, 20–30% from protein and the rest from carbohydrates (40–60 g/day) [24]. It is limited to foods with a glycaemic index less than 50, such as lentils and whole-grain high-fibre bread, to minimise the rise in blood glucose with meals, based on the observation that acute elevations in blood glucose are associated with sharp reductions in blood ketone levels [18, 24]. It has been shown to induce ketosis above the normal serum ketone range, with associated reductions in seizure episodes in patients with epilepsy [24].

More recently, a distinct “modified ketogenic diet” has been described that combines elements of all four ketogenic diet variants described so far, with targets of approximately 60–80% of energy from fat (combining medium- and long-chain triglycerides), 5% of energy or 15–30 g/day of carbohydrates, and ad libitum protein [25].

#### Preclinical Studies of Ketogenic Diets

**Fig. 1:**
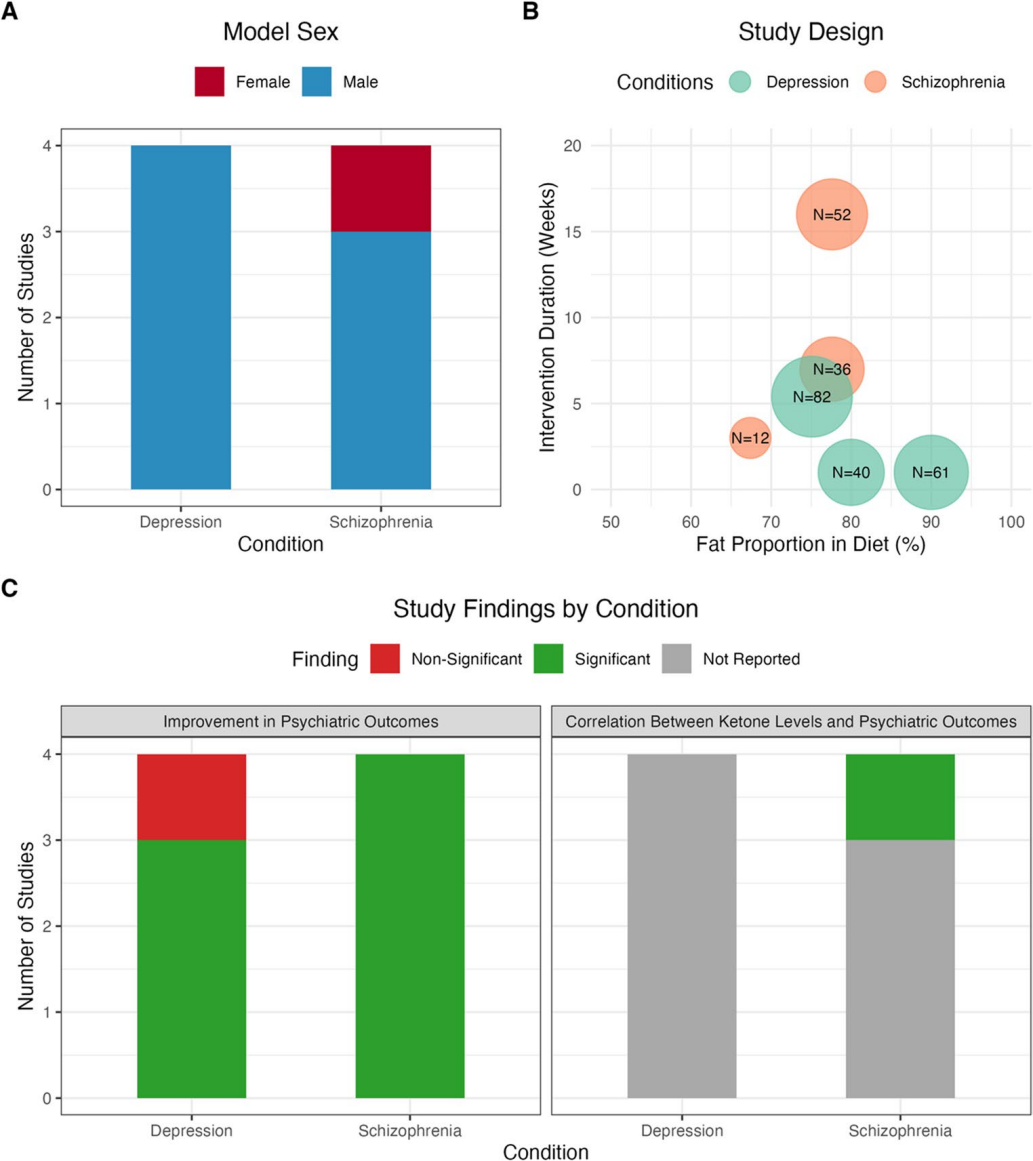
Summary of the eight preclinical studies evaluating ketogenic diet efficacy in models of severe mental illness. **A**. Model Sex, Sex distribution of the animals used in the reviewed studies, none of the identified studies used a bipolar model. **B**. Study design, study details by condition modelled including sample size, intervention duration, and proportion of fat in diet. Six of the eight studies are represented; of the rest, one did not report sample size and one did not report dietary macronutrient composition, precluding their inclusion in this panel. **C**. Study findings by condition, including number of studies reporting significant improvements in psychiatric symptom correlates by condition, as well as proportion of studies showing a correlation between ketone levels and treatment outcomes by condition

Preclinical studies have reported some evidence that commercially prepared ketogenic diets of varying macronutrient composition may improve correlates of severe mental illness symptoms in rodent models (Fig. 1).

DBA2 mice are genetic models that show impaired hippocampal gating of the P20/N40 wave, which is the mouse correlate of the P50, a positive event-related potential observed on hippocampal electroencephalograms in response to paired auditory stimuli in patients with schizophrenia [26, 27]. A ketogenic diet administered over three weeks was shown to significantly suppress the P20/N40 response in male DBA/2 mice with ketone levels above 0.5mM compared to those with lower ketone levels [28]. P20/N40 suppression was also positively correlated with ketone levels, suggesting a dose-response relationship between ketosis and restored sensory gating. However, the study did not have a control group precluding firm conclusions that the observed effects were entirely due to the ketosis.

Multiple preclinical studies have similarly demonstrated a positive effect of ketogenic diets on sensory gating observed as exaggerated motor responses to the second of a pair of auditory stimuli using another schizophrenia model: an acute N-methyl D-Aspartate (NMDA) receptor hypofunction model developed by administering the NMDA receptor antagonist, dizocilpine [29]. A ketogenic diet administered for seven weeks prevented dizocilpine-induced impairment of prepulse inhibition of the startle response in male mice [30], producing similar effects as the antipsychotic olanzapine. A similar study done over three weeks reported additional benefits, including reduced social and working memory deficits, and lesser psychomotor hyperactivity and stereotyped behaviour in male mice [31]. In both cases, ketone (beta-hydroxybutyrate) levels measured prior to behavioural testing were significantly greater in mice receiving ketogenic diets than in control mice (~ 5mM vs. 2mM, *p* < 0.001 [30]; 1mM vs. ~ 0.6mM, *p* < 0.001 [31]). The higher ketone levels in both mouse groups in the seven-week study may be due to the longer duration of dietary exposure compared to the three-week study. Dose-response relationships were not explored in either study.

These results offer preclinical evidence suggesting potential benefits of ketogenic diets in two validated endophenotypes of schizophrenia that converge on impaired sensory filtering. However, questions remain about whether these findings would be replicated in patients with schizophrenia and the extent of associated clinical benefits. Furthermore, ketone levels were measured only once in the dizocilpine studies, precluding the assessment of dose-response relationships observed in the DBA/2 mice. Additionally, these studies primarily used male mice precluding generalisation across sexes (Fig. 1A). While a study done in female mice showed beneficial effects of a ketogenic diet on prepulse inhibition, ketone levels were not reported [32].

Some preclinical studies have also examined the effects of ketogenic diets on depression-related behavioural phenotypes in rodent models of depression (Supplementary Table 1). Two studies found reduced immobility duration in forced swim and tail suspension tests, increased social interaction times and increased sucrose preference in chronic social defeat male mice fed a ketogenic diet [33, 34], with one of the studies finding these effects were specific to stress-susceptible mice and absent in resilient or unstressed controls [33]. In the latter study by Guan et al., beta-hydroxybutyrate levels were significantly higher in the ketogenic-diet-fed mice prior to behavioural testing and were associated with reduced pro-inflammatory markers, attenuated microglial activation, and reduced lateral habenular excitability in stressed or lipopolysaccharide-injected mice but not controls [33]. Liang et al. also demonstrated similar anti-inflammatory effects in mice, however, ketone levels were not reported [34]. Another study [35] found that a ketogenic diet improved microglial homeostasis in healthy mice but did not demonstrate differential anti-inflammatory effects in stressed versus healthy and resilient mice fed a ketogenic diet compared to those fed a control diet, in contrast to the Guan et al. findings [33]. Notably, the authors examined the hippocampus rather than the lateral habenula which may partly account for the divergent results. Ketogenic diets were also found to reduce immobility duration in healthy male Wistar rats subjected to the forced swim test after seven days on the diet [36]. Although beta-hydroxybutyrate levels were significantly higher in ketogenic-diet-fed rats in this study, ketone levels were relatively low in both groups and were measured after (rather than before) behavioural testing [36].

Limitations of these preclinical depression studies include small sample sizes used for subgroup analyses, limited assessment of long-term effects, no inclusion of female rodents, and the absence of dose-response analyses (Fig. 1C). Additionally, Murphy et al. used otherwise healthy rats subjected to the acute stress of the forced swim test limiting comparison with studies done in chronic stress models of depression [36].

Prenatal exposure to a ketogenic diet comprising 67.1% fat, 15.3% protein and 9.2% carbohydrates has also been found to be associated with significantly reduced immobility duration in the forced swim test and increased sociability in offspring of ketogenic-diet-exposed mice compared to offspring of mice fed a standard diet, suggesting that ketogenic diets may have developmental effects on depressive symptom correlates [37]. However, the mechanistic link between the exposures and behavioural changes in offspring are unclear and temporal differences between rodent and human gestation periods mean that it is difficult to resolve the window in which such exposures may result in similar effects in humans without jeopardising foetal development.

No preclinical studies meeting our inclusion criteria examined ketogenic diets in animal models of bipolar disorder.

#### Clinical Trials of Ketogenic Diets

**Fig. 2:**
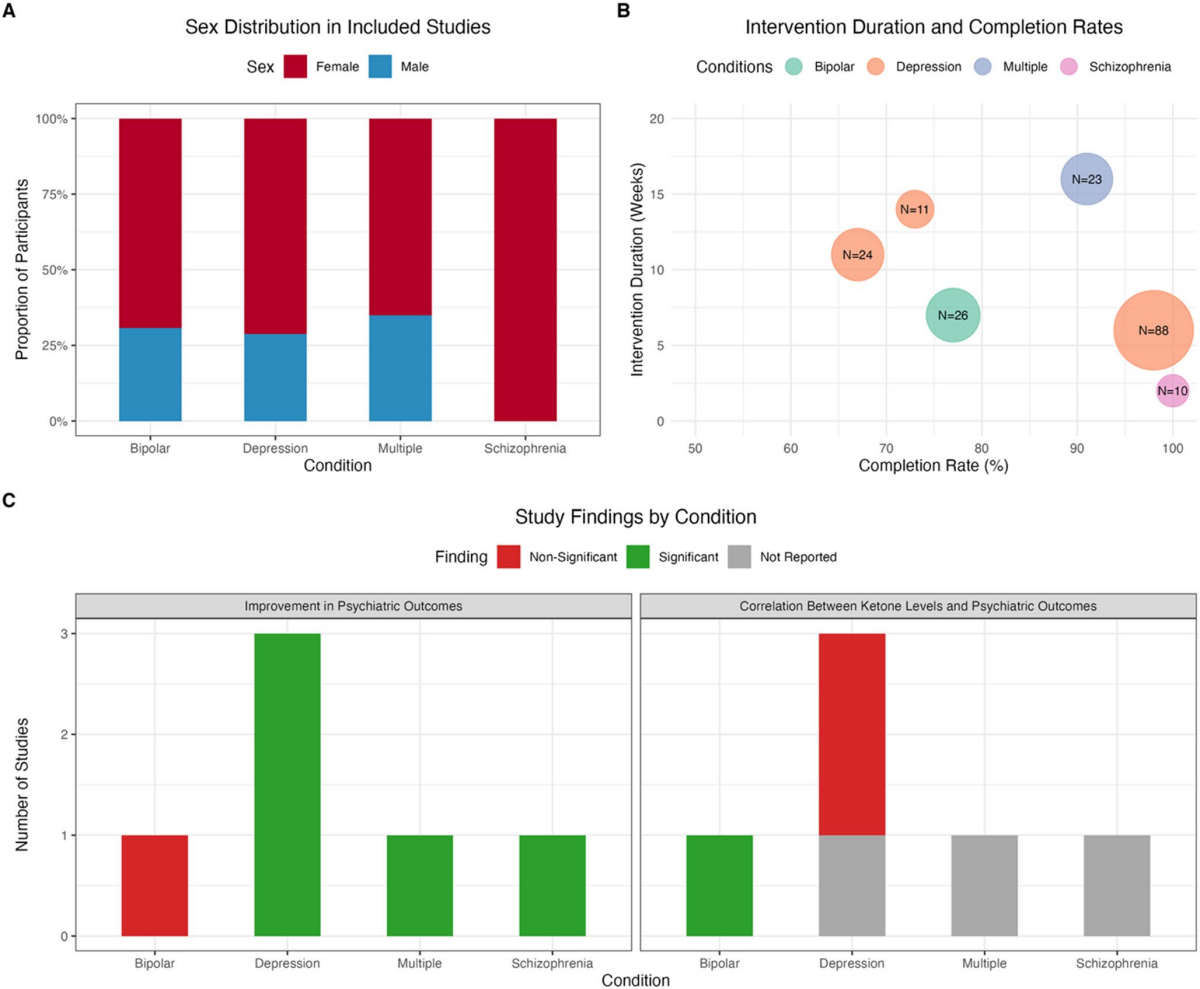
Summary of the six included clinical trials evaluating ketogenic diets in severe mental illness. **A**. Sex distribution in included studies by condition; ‘Multiple’ denotes a combined schizophrenia and bipolar disorder cohort for which condition-specific analyses were not reported [40]. **B**. Intervention duration and completion rates. Each dot represents a study. **C**. Study findings by condition. Improvement in psychiatric outcomes. Chart shows whether or not there were significant changes in primary psychiatric outcomes in included studies by condition. The stand-alone bipolar trial examined whether there were significant changes in depressive or manic symptoms of patients who were euthymic at baseline, hence the non-significant result reflects that participants remained euthymic

A summary of the included studies is provided in Fig. 2 with detailed study descriptions and findings available in Supplementary Table 2. Most of the trials did not report using any of the specific named ketogenic diet variants of described above. One of the trials used a modified ketogenic diet with macronutrient compositions that largely corresponded to the distinct modified ketogenic diet approach [38, 39].

We identified one uncontrolled pilot trial evaluating a ketogenic diet in patients with schizophrenia in which ketone levels were reported. The diet comprised 60% fat, 30% protein and 10% carbohydrates and was tested in a combined population of patients with schizophrenia, schizoaffective disorder or bipolar disorder [40]. Ketone levels were measured at least once a week for the 4-month duration of the study, and 20 of 21 study completers had ketone levels above 0.5mM at ³ 60% of measurement time points. The authors reported significant improvements in mean Clinical Global Impression of severity of mental illness scores, Manchester Short Assessment of Quality of Life scores, serum triglycerides, haemoglobin A1c, and homeostatic model assessment of insulin resistance scores across the entire cohort. PHQ-9 depression scores improved significantly within the adherent and semi-adherent subgroups but not for the cohort overall, and among the five participants with schizophrenia, Brief Psychiatric Rating Scale scores fell significantly. However, these findings occurred within the context of significant reduction in mean body weight and the authors did not examine whether the degree of ketosis was associated with the extent of improvement in psychiatric outcomes. A previous study also showed improved Beckomberga scale symptom ratings in patients with treatment-resistant schizophrenia, however the ketogenic diet composition and participants’ ketone levels were not reported [41].

To the best of our knowledge, only one randomised controlled trial of ketogenic diets has been conducted in major depressive disorder. The study tested a ketogenic diet variant in 88 patients with treatment-resistant depression and found no significant difference in improvements in mean Patient Health Questionnaire-9 (PHQ-9) scores from baseline between the ketogenic diet group and the phytochemical diet group at six weeks (*p* = 0.05), or at 12 weeks (*p* = 0.10) [42]. Subgroup analyses indicated a substantially larger treatment effect in participants with severe depression (PHQ-9, 20–27) than in those with moderate depression (PHQ-9, 15–19), in whom no benefit was observed. Exploratory analyses showed no significant association between ketone concentration and PHQ-9 score changes, and participants with mean ketone levels below 1.5mM showed greater PHQ-9 improvements than those with levels at or above this threshold, with a similar pattern observed at the 4mM threshold, raising questions about the role of ketosis in inducing the changes observed. Additionally, the study was designed to optimise adherence by providing prepared meals during the intervention period, but ketogenic diet uptake dropped steeply within six weeks post-intervention, highlighting the importance of ongoing support for maintaining adherence. Changes in antidepressant use were permitted in both groups but were not monitored or reported.

Uncontrolled pilot trials have also suggested therapeutic benefits of ketogenic diets in depression, however as detailed in Supplementary Table 2, they were limited by small sample sizes, poor diversity of their study populations, and high drop-out rates, and were possibly confounded by expectation bias and concurrent or changing psychiatric treatment [43, 44]. Notably, depression improvement in one of these trials was not associated with the degree of ketosis achieved [43], raising similar questions as the randomised controlled trial (described above) about the therapeutic role of ketosis (Fig. 2C).

There were no randomised controlled trials of ketogenic diets in patients with bipolar disorder, however, an uncontrolled pilot study has tested the modified ketogenic diet in patients with bipolar disorder who were euthymic [38, 39]. Nineteen of 20 participants who completed baseline and follow-up assessments showed good adherence, with 91% of daily blood ketone readings indicating ketosis (≥ 0.5mM). More than half of the study completers lost at least 5% of their initial body weight. Daily blood ketone readings positively correlated with self-rated mood and energy levels and negatively correlated with impulsivity and anxiety. Glutamate and glutamine concentrations in the anterior and posterior cingulate cortices decreased [38], aligning with prior evidence of glutamate elevation in the anterior cingulate cortex in bipolar depression [45] as well as existing literature linking glutamine and glutamate reductions with antidepressant effects [46, 47]. However, the findings of this study may not be generalisable to patients with bipolar who are not euthymic.

### Exogenous Ketone Supplements

Exogenous ketone supplements are salts or esters of the ketone body, beta-hydroxybutyrate, developed to reproduce the therapeutic benefits associated with ketogenic diets, without dietary restriction. The salts consist of beta-hydroxybutyrate bound to minerals such as sodium, potassium or magnesium, while the esters typically include precursor molecules such as 1,3-butanediol [48]. Both types of exogenous ketone supplements are available as oral formulations.

Evidence from a systematic review of 43 clinical trials (*n* = 586) across healthy participants and those with diabetes, overweight, or obesity, suggests that ketone supplements significantly increase blood concentrations of beta-hydroxybutyrate and reduce blood glucose [49]. However, heterogeneity between included studies was high, suggesting variable ketosis-inducing effects of ketone supplements across populations. Ketone monoesters also produced significantly greater ketosis-inducing effects (mean difference 2.57mM vs. 0.5mM; *p* < 0.001) and fewer reported side effects than ketone salts, suggesting that different ketone supplement formulations may have varying efficacy and tolerability profiles.

Although our search did not yield any clinical trials assessing the effects of exogenous ketone supplements in patients with severe mental illness, several preclinical studies have reported antidepressant-like effects of beta-hydroxybutyrate administration in the chronic unpredictable stress mouse model of depression. Repeated subcutaneous [50] or cerebral [51] injections of beta-hydroxybutyrate esters significantly reduced stress-induced increases in time spent immobile in the forced swim test. The rise of pro-inflammatory markers, including tumour necrosis factor-alpha, interleukin-6, and interleukin-1 beta, induced by chronic unpredictable stress, was also attenuated by ketone esters [51]. Similarly, pre-treatment with ketone esters in drinking water for 3 days has been reported to strongly diminish the lipopolysaccharide-induced expression of the above pro-inflammatory markers in mouse brains compared to controls [52].

One study in the dizocilpine-induced NMDA hypofunction model of schizophrenia [53] found that acute administration of 10mM or 20mM of beta-hydroxybutyrate significantly increased ketones above levels observed in saline-treated mice (2.08mM ± 0.38 and 6.95mM ± 0.61 vs. 0.56mM ± 0.05, *p* < 0.05) and prevented the attenuation of prepulse inhibition of the startle response observed in controls, aligning with earlier findings from testing ketogenic diets in the same mouse model.

These findings, when taken with earlier studies involving ketogenic diets, suggest that attenuating inflammation might be one of the mechanisms through which ketogenic diets and other ketosis-inducing interventions exert their therapeutic effects in these conditions. Nonetheless, studies are required to test which ketone supplements reliably and safely induce ketosis in humans, what factors determine whether ketosis develops following administration of a ketone supplement, and whether these supplements have beneficial effects in clinical populations with severe mental illness.

### Oral Medium-Chain Triglyceride Compounds

Medium-chain triglycerides are esters of saturated medium-chain fatty acids (comprising 6–12 carbon atoms) which are obtained by hydrolysing coconut oils and are more rapidly hydrolysed than long-chain fatty acids that comprise the triglycerides of the classic ketogenic diet [54]. Evidence from preclinical studies suggests that medium-chain triglycerides rapidly induce ketosis [55] and to a greater degree than comparable amounts of long-chain triglycerides [56].

Our literature search did not reveal any studies investigating the effects of medium chain triglyceride supplementation in severe mental illness. However, evidence from preclinical and clinical studies suggest that medium chain triglycerides improve cognition. For example, chronic dietary supplementation with medium-chain triglycerides improved working memory in Wistar rats [57]. A systematic review and meta-analysis reported improved mini mental state examination scores in persons with Alzheimer’s disease [58], although the specific cognitive domains affected were not stated. Other studies reported significantly better recall in older adults without dementia [59] and better working memory in younger healthy adults [60] who received medium-chain triglyceride diet supplementation than in those who received a long chain triglyceride supplement for 3 months and 4 weeks respectively. Medium chain triglyceride supplementation also appears to produce metabolic effects in humans similar to those associated with ketogenic diets such as significant reductions in body weight, BMI and waist circumference [61].

These findings may be relevant for severe mental illnesses such as schizophrenia [62] and depression [63] where cognitive deficits may impair patients’ functioning and quality of life and metabolic dysfunction contributes significantly to their associated morbidity. However, randomised controlled trials in populations with severe mental illnesses are required to ascertain these cognitive and metabolic effects.

### Fasting and Time-Restricted Eating

Historically, ketogenic diets were devised as a way to mimic the apparent therapeutic effect of fasting on epilepsy [64]. However, we did not find any clinical trials investigating the benefits of fasting in populations with severe mental illness and studies examining mental health symptoms in non-psychiatric populations have not measured ketone levels, precluding any direct test of whether ketosis underlies the effects observed. A non-randomised controlled trial done in 36 healthy volunteers undergoing eight weeks of weekly 24-hour fasting found no between-group difference in mean Hospital Anxiety and Depression Scale (HADS) and the World Health Organisation-5 well-being index scores of those in the fasting versus the non-fasting group in the immediate aftermath of the intervention, despite a significantly greater reduction in body fat mass in the fasting group [65]. Another non-randomised trial in healthy females and females with obesity found that a single 10-hour daytime fast significantly worsened mood in participants with obesity but not in those without [66], suggesting that the psychological impact of fasting may be moderated by adiposity rather than reflecting a consistent ketosis-related benefit.

The cited studies used fasting protocols varying in duration of daily abstinence and fasting periods limiting comparability between studies. They also lacked objective means of assessing fasting compliance beyond participant self-reports and were not designed to ascertain the role of ketosis in the effects observed. This is particularly relevant because preclinical studies have shown that fasting has physiologic effects beyond ketosis, including stimulating mitochondrial biogenesis and cellular repair processes by modulating the abundance of energy carriers (such as nicotinamide adenine dinucleotide) which in turn activate a variety of transcription factors [67] with several downstream effects.

Circadian dysfunction is well-documented in severe mental illness [68] and is associated with abnormal patterns of sleep, eating, and metabolism. Time-restricted eating, which consolidates food intake within a defined daily window without necessarily reducing caloric intake, represents a structured dietary approach that aims to align eating patterns with circadian biology. Emerging evidence suggests that meal timing independently influences metabolic outcomes. Later dinner time and longer intervals between breakfast and lunch are associated with greater waist circumference after adjusting for age, sex, and sleep duration [69]. Late main meal eaters also show significantly reduced glucose tolerance, less weight loss and slower weight loss rate, compared to early main meal eaters despite similar energy intake, dietary composition, and estimated energy expenditure [70]. Additionally, there is evidence suggesting that time-restricted eating could improve mental health, with a crossover trial in patients with diabetes showing that time-restricted eating was associated with significantly lower Beck depression inventory scores [71].

Although the cited studies did not measure ketone levels, their observed effects parallel the mental health and metabolic benefits associated with ketogenic diets, and a separate randomised controlled trial has shown that time-restricted eating induces modest ketosis in humans [72]. The study compared early time-restricted eating (8am-4pm), late time-restricted eating (2pm-10pm), modified alternate-day fasting, and a very low-carbohydrate ketogenic diet against a calorie-restricted Mediterranean diet in 160 adults with obesity over 3 months. The Mediterranean diet produced significantly lower ketone levels than all other interventions, however, specific figures were not reported. This suggests that the metabolic and mental health effects of time-restricted eating may be partly mediated by ketosis, however this remains to be directly demonstrated, particularly in populations with severe mental illness.

## Is Ketosis the Primary Mediator?

Several preclinical studies have shown significant associations between ketone levels and improvements in schizophrenia- and depression-related deficits but without demonstrating dose-response relationships. One preclinical study of a ketogenic diet in DBA/2 mice showed significantly reduced impairments in sensory gating and a dose-response relationship between ketone levels and sensory filtering in mice fed a ketogenic diet for three weeks, however, there was no control group, meaning the observed effects cannot be reliably attributed to the ketosis-inducing intervention specifically.

Most of the evidence from clinical interventional studies has come from early, non-randomised, uncontrolled trials which have a number of important limitations. Firstly, they lack control groups with comparator diets and have been conducted in the setting of concurrent psychiatric care, meaning that the observed effects may not be due to the dietary interventions alone. Additionally, as the pilot trials contain only one arm, questionnaire raters and participants are functionally unblinded making the results prone to expectation bias [73].

Furthermore, although a pilot trial of a ketogenic diet in euthymic patients with bipolar disorder found a positive correlation between ketone levels and reduced impulsivity and increased energy, most of the study participants experienced significant weight loss [38], similar to the combined schizophrenia and bipolar pilot study [40]. Weight loss has been associated with improvement in overall psychological well-being in the absence of any specific dietary intervention [74, 75], suggesting that the mood effects reported in these studies could partly be explained by the effects of weight loss rather than ketosis specifically. Additionally, the structured nature of dietary intervention trials involving regular clinical contact, and monitoring mood and ketone levels may have non-specific intervention effects that contribute to mood improvement.

The only randomised controlled trial of a ketogenic diet in a severe mental illness population partially addressed these concerns through its controlled design and weight-stabilisation protocol [42], but questions about the role of ketosis remain unresolved. The ketogenic diet group showed only marginally greater improvements in PHQ-9 scores (2.18 points) than in controls, which did not reach statistical significance (*p* = 0.05). Participants in the phytochemical diet control group showed a mean improvement of 8.3 points in PHQ-9 scores which was substantially greater than the additional benefit attributable to the ketogenic diet, suggesting that factors common to both intervention groups such as regular clinical contact and dietary support, may have accounted for most of the observed mood improvements. The absence of relapse to pre-intervention depression levels after support was withdrawn in both groups further raises the possibility that improvement was not entirely related to the dietary components of either intervention. Additionally, ketone levels were not measured in the control group, meaning the trial cannot definitively establish whether the presence or absence of ketosis distinguished the two arms. Furthermore, within-group analyses for the ketogenic diet group showed that ketone concentrations were not associated with PHQ-9 scores, and subgroup analyses revealed greater improvements in patients with ketone levels below rather than above set thresholds (1.5mM and 4.0mM), further raising questions about the extent to which ketosis contributes to improved symptoms. This apparent dissociation between ketosis and symptom improvement echoes the finding from an uncontrolled pilot trial in depression, in which ketone levels were similarly unrelated to the degree of clinical benefit observed [43].

Several studies have demonstrated a relationship between the gut microbiome and brain function [76, 77], and abnormalities in the gut microbiome are associated with increased risk of severe mental illnesses such as schizophrenia and major depressive disorder [78–80]. Ketogenic diets have been shown to alter the gut microbiome [81, 82], and a recent preclinical study revealed that faecal microbial transplantation from ketogenic-diet-fed mice to standard-diet-fed mice mitigated dizocilpine-induced impairments in sensory gating [83], similar to the effect of ketogenic diets reported in earlier studies. This raises the possibility that some effects of ketogenic diets on psychiatric symptoms may be mediated through microbiome changes rather than ketosis directly, however only one of the reviewed trials examined microbiome changes, with findings to be reported in a future publication [42].

Taken together, current evidence does not establish ketosis as the primary mediator of the psychiatric benefits associated with ketosis-inducing interventions. The clearest signal of a ketosis-outcome relationship comes from an uncontrolled preclinical study, while the only randomised controlled trial found no such association, and several plausible non-ketosis mechanisms remain unresolved.

## Clinical Considerations

Although no ketosis-inducing intervention is currently approved or recommended as a treatment for severe mental illness, clinicians should be equipped to discuss the available evidence with patients who ask about these approaches and advise them accordingly.

Ketogenic diets should be avoided in patients with type 1 diabetes or other insulin-deficient states due to the risk of euglycemic ketoacidosis [84]. Caution should also be exercised in recommending a ketogenic diet to patients with type 2 diabetes who are receiving sodium glucose transporter (SGLT2) inhibitors as the combination synergistically increases the risk of euglycemic ketoacidosis [85], as demonstrated in the recent pilot study in patients with bipolar disorder [39]. Given the increased risk of dyslipidaemia with ketogenic diet therapy [86], all patients receiving the intervention might benefit from periodic lipid profile (serum concentrations of total cholesterol, low-density lipoprotein, high-density lipoprotein, and triglycerides) and heart health assessments such as echocardiograms and electrocardiograms.

Folate deficits have been reported in pregnant patients given a restricted carbohydrate diet, along with a significantly greater likelihood of having offspring with neural tube defects [87], suggesting that ketogenic diets should be avoided in this population. Non-pregnant patients who intend to commence a ketogenic diet should be advised to take readily available micronutrient supplements during the intervention period to meet their daily requirements [88].

Clinicians should also be aware of potential drug-specific interactions relevant to patients considering a ketogenic diet. Caution should be exercised in recommending a ketogenic diet to patients with schizophrenia who are receiving clozapine, as constipation, a frequent side-effect of ketogenic diets [88], occurs in up to 80% of patients receiving the drug [89].

Finally, clinicians should be clear with patients that the evidence base for ketosis-inducing interventions other than ketogenic diets is considerably weaker. Exogenous ketone supplements and oral medium-chain triglyceride compounds have not been tested in populations with severe mental illness and fasting and time-restricted eating studies have produced inconsistent results without measuring whether ketosis was achieved. Patients should thus be advised accordingly when discussing these options.

## Considerations for Future Research

Preclinical studies are required to further unravel the range of effects of ketogenic diets and other ketosis-inducing interventions on correlates of symptoms of severe mental illness. Specifically, more endophenotypes of psychiatric deficits need to be tested across conditions. For example, current schizophrenia animal studies have largely focused on defective sensory gating and positive symptom correlates, whereas negative symptoms such as amotivation and social withdrawal [90] as well as cognitive symptoms such as working memory and processing speed deficits [91] contribute significantly to the morbidity associated with the condition and are not amenable to current treatments. Notably, no preclinical studies meeting our inclusion criteria examined bipolar disorder. Studies testing bipolar models, and those utilising schizophrenia models that recapitulate a broader range of symptoms, as well as those that incorporate their commonly co-occurring metabolic abnormalities [92–96] may provide more insight into the broader psychiatric and metabolic effects of ketosis-inducing interventions.

More randomised controlled trials evaluating ketogenic diets for severe mental illness in clinical populations are also required. So far, only one such trial has been reported, which was done in patients with treatment-resistant depression. Several randomised controlled trials appear to be underway, including in bipolar disorder [97–99] and depression [100]. Future trials should be designed to isolate the role of ketosis specifically. Although the existing randomised controlled trial controlled for weight loss and matched support across arms, most other clinical evidence in this field comes from uncontrolled pilot trials; replicating these controlled design features, alongside using non-ketogenic comparator diets that are equally demanding to implement and measuring ketosis frequently to assess dose-response relationships, remain priorities for future work. Additionally, the uncontrolled and controlled clinical trials mostly utilised unique ketogenic diet variants that did not correspond to historical formulations and varied in the amount of detail provided about the macronutrient compositions of the diets used (Supplementary Table 2). Only the randomised controlled trial reported an explicit ratio of fat-to-carbohydrate-and-protein (in the study protocol) [42]. Two trials adjusted macronutrient percentages as the study progressed, with one of the studies varying the diet based on a target blood ketone level of 1-4mM [39] while the other used preplanned phased macronutrient adjustments [44]. In order to facilitate comparison across studies and aid reproducibility of research findings, future trials should report fat-to-carbohydrate-and-protein ratios and how they compare with previous studies, as well as their justification for any deviations from recognised ketogenic diet variants.

Future studies also need to assess adherence to ketogenic diets in patients without access to prepared meals and the mechanisms by which ketogenic diets exert their apparent effects such as the anti-inflammatory changes observed in preclinical depression models, neurotransmitter modulations reported in the recent bipolar disorder pilot trial or microbiome effects reported preclinically. It is also important to investigate the duration of therapeutic benefits of ketogenic diets in both mental and metabolic health following discontinuation, whether they reduce the need for psychotropic medications, and whether their effectiveness varies with illness severity (as suggested by the randomised controlled trial in depression).

Additionally, although evidence from preclinical studies suggests that other ketosis-inducing interventions may be associated with psychiatric benefits similar to those reported with the use of ketogenic diets, these interventions have yet to be tested in clinical populations with severe mental illness. Studies are required to ascertain which exogenous ketone formulations induce ketosis safely and reliably in humans and at what dose and to investigate whether oral medium chain triglyceride supplements produce cognitive benefits in severe mental illness similar to those observed in preclinical studies and non-psychiatric populations. Ultimately, trials comparing these interventions directly with ketogenic diets in populations with severe mental illness are needed to determine whether these less restrictive approaches produce comparable psychiatric and metabolic benefits to ketogenic diets. If they do, they may offer more accessible and sustainable alternatives for patients who cannot tolerate the dietary demands of a ketogenic diet, potentially improving the uptake of ketosis-based treatments in clinical practice.

Fasting and time-restricted eating have not yet been tested for their effects on severe mental illness in randomised controlled trials. Existing clinical trials of fasting in non-psychiatric populations have not measured ketone levels, leaving it unclear whether their reported mental health effects are mediated by ketosis. Randomised controlled trials are needed to directly assess the effects of fasting and time-restricted eating on severe mental illness; to establish the speed and extent to which they induce ketosis in these populations; and to clarify the mechanisms — common or distinct from those of ketogenic diets — by which any observed benefits occur.

## Conclusions

While there is some evidence that ketosis inducing interventions have mental health benefits, the role of ketosis in mediating these effects remains unclear. Several plausible alternative explanations beyond ketosis itself exist, highlighting the need for well-controlled studies designed to isolate the role of ketosis. Where guidance is sought by patients, clinicians should note that the clinical evidence for severe mental illness remains limited to ketogenic diets specifically, and highlight the need for more studies before evidence-based recommendations can be made.

## Supplementary Information

Below is the link to the electronic supplementary material.

Supplementary Material 1

## Data Availability

No datasets were generated or analysed during the current study.
