# Supplementary material for: Ketosis as a Treatment for Severe Mental Illness: A Critical Review of Preclinical and Clinical Data
Source: Curr Treat Options Psychiatry. 2026 Aug 17;13(1):20. doi: 10.1007/s40501-026-00394-z (PMC13481580; doi:10.1007/s40501-026-00394-z)
Supplement: Supplementary file 1 [file 40501_2026_394_MOESM1_ESM.docx]

***Supplementary Table 1****: Preclinical studies investigating ketogenic diets in animal models of severe mental illness*

| **Lead Author, Year** | **Animal model** | **Sample size** | **Intervention** | **Duration** | **Key findings** | **Limitations** |
| --- | --- | --- | --- | --- | --- | --- |
| **Schizophrenia models** | | | | | | |
| Kraeuter, 2019 [32] | Female C57BL/6 mice.  Acute NMDA receptor hypofunction model of schizophrenia (dizocilpine-induced) | N = 52 | Ketogenic diet (77.6% fat, 9.5% protein, 9.4% carbohydrate) vs. standard mouse diet for 4 months, then randomised to olanzapine (2 mg/kg intraperitoneally twice daily) or saline for 8 weeks (2×2 factorial design: n=13/group).  Dizocilpine (0.25 mg/kg intraperitoneally) administered 30 minutes before prepulse inhibition testing. | 4 months | Ketogenic diet alone (p=0.002), olanzapine alone (p<0.001), and combination (p=0.016) each prevented dizocilpine-induced prepulse inhibition disruption  Ketogenic diet reduced startle amplitude (p=0.003) | Acute pharmacological model.  Limited to prepulse inhibition endophenotype  Ketone levels not reported.  Ketogenic diet commenced before deficit induced as opposed to within the setting of established deficits. |
| Kraeuter, 2019 [30] | Male C57BL/6 mice  Acute dizocilpine-induced NMDA receptor hypofunction schizophrenia model | N = 36 | Ketogenic diet (77.6% fat, 9.5% protein, 9.4% carbohydrates) vs. Standard mouse diet.  Dizocilpine (0.25 mg/kg intraperitoneally) vs. saline in crossover design administered 30 minutes before prepulse inhibition testing. | 7 weeks (behavioural tests at weeks 3 and 7) | Week 7 beta-hydroxybutyrate levels: ~5mM in ketogenic diet mice vs 2mM in standard diet mice, p<0.001  Ketogenic diet prevented dizocilpine-induced prepulse inhibition deficits at both timepoints independent of energy deficits.  Ketogenic diet mice had lower body weight throughout (all weeks p<0.001) | Acute pharmacological model.  Limited to prepulse inhibition endophenotype  Ketogenic diet commenced before deficit induced as opposed to within the setting of established deficits. |
| Kraeuter, 2015 [31] | Male C57BL/6 mice; acute dizocilpine-induced NMDA receptor hypofunction schizophrenia model | Not reported | Ketogenic diet (77.6% fat; 9.5% protein; 9.4% carbohydrates) or a standard mouse diet.  Dizocilpine administered intraperitoneally at 0.1, 0.2 or 0.4mg/kg vs saline 30 minutes before testing | 3 weeks | Week 3 Beta-hydroxybutyrate levels: 1mM in ketogenic diet mice vs ~0.6mM in standard diet mice, p<0.001  Resolved working memory and social interaction deficits  Attenuated psychomotor hyperactivity and reduced stereotyped behaviour  Lower body weight and glucose levels and significantly higher beta-hydroxybutyrate levels compared to standard diet mice. | Sample size not reported.  Acute pharmacological model does not accurately model chronic progressive neurobiology of schizophrenia.  Intervention was implemented prior to pharmacologic insult so study tested prevention rather than treatment of prior established deficits.  No mechanistic data.  Unclear if effects persist after diet cessation. |
| Tregellas, 2015 [28] | DBA/2 male mice (genetic model of auditory gating deficits in schizophrenia | N = 12 | Ketogenic diet (0.6% carbohydrate, high fat) administered to all animals. No control diet group. | 3 weeks | Higher blood ketone levels were associated with lower test-to-conditioning stimulus response (TC) ratios, reflecting improved sensory gating (r = −0.679, p = 0.008)  High-ketosis group had significantly lower TC ratio than low-ketosis group (p<0.001)  S2 (test) amplitude was significantly reduced in the high-ketosis group (p = 0.021) | No control group or comparator diet |
| **Depression models** | | | | | | |
| Liang, 2025  [34] | Male C57BL/6 mice.  Chronic unpredictable mild stress depression model | N = 48 | Ketogenic diet (macronutrient composition not reported) vs control diet  Chronic unpredictable mild stress and (unchallenged) control group each subdivided into ketogenic diet and control groups | ~8 weeks | Ketogenic diet significantly reduced immobility times in the forced swim and tail suspension tests and increased sucrose preference in the chronic unpredictable mild stress mice but not control mice.  Ketogenic diet significantly reduced the levels of inflammatory markers including interleukin 1-beta, tumour necrosis factor-alpha, and cyclo-oxygenase 2, and attenuated microglial and astrocyte activation in the hippocampus of chronic unpredictable mild stress mice but not controls. | Ketone levels not reported  Composition of ketogenic and control diets used not reported |
| González Ibáñez, 2023 [35] | Male C57BL/6J mice  Repeated social defeat model of depression | N = 82 | Ketogenic diet (75.1% fat; 8.6% protein; 3.2% carbohydrate; 4.8% fibre) vs control diet (18% fat; 58% carbohydrate; 24% protein) | 4 weeks prior to social defeat exposures and during the 10 days of social defeat exposures | Significantly higher plasma beta-hydroxybutyrate levels in ketogenic-diet-fed mice compared to control diet mice prior to and after social defeat exposures (day 12).  Ketogenic diet tended to increase the proportion of stress-resistant mice following repeated social defeat (57% resistant vs 36% on control diet), though this did not reach statistical significance (p=0.072)  Ketogenic diet modified microglial morphology at baseline in unstressed animals, with larger soma and greater arborization area in ketogenic diet controls compared to control diet controls, but did not significantly alter microglial morphology specifically in susceptible mice. | Diet started prior to social defeat exposures rather than in the setting of established deficits, as in typical clinical scenarios. |
| Yan Fei Guan, 2020 [33] | Male C57BL/6J mice  Repeated social defeat stress depression model (susceptible and resilient mice and controls)  Lipopolysaccharide depression model | N = 61  Susceptible group: n = 21  Resilient group: n = 20  Control group: n = 20 | Ketogenic diet (90% fat; 10% protein) vs control diet (10% fat; 80% carbohydrate; 10% protein)  3x2 factorial design (susceptible, resilient, and control mice groups each subdivided into control diet and ketogenic diet subgroups) | 7 days | Significantly higher beta-hydroxybutyrate levels in ketogenic diet mice vs control mice one week after commencing dietary interventions.  Ketogenic diet significantly increased social interaction ratio, and reduced immobility times in the forced swim and tail suspension tests in susceptible mice but not in control or resilient mice and in lipopolysaccharide-injected mice but not in vehicle-injected mice  Ketogenic diet reduced lateral habenular nucleus firing frequency, microglial cell body volume and total process length, tumour necrosis factor-alpha, interleukin 1-beta, and interleukin-6 mRNA levels in susceptible repeated social defeat mice and lipopolysaccharide-injected mice but not others  Ketogenic diet increased TREM-2 expression in susceptible repeated social defeat stress and lipopolysaccharide-injected mice | Small sample size for subgroup analyses. For example, n=3 for microglial total process length and mRNA levels of pro-inflammatory markers. |
| Murphy, 2004 [36] | Male Wistar rats; Acute behavioural despair (forced swim) | N = 40 | Ketogenic diet (fat and carbohydrate + protein in 4:1 ratio) vs control diet (fat and carbohydrate + protein in 0.082:1 ratio).  Both groups subjected to the forced swim test | 7 days | Significantly greater mean beta-hydroxybutyrate levels in ketogenic diet-fed vs control diet-fed mice prior to behavioural testing (0.579mM ± 0.177mM vs 0.304mM ± 0.104mM, p<0.001)  Significantly less time spent immobile in ketogenic diet group compared to control group | Low ketone levels in both groups.  Only 12 of 20 animals tested for ketosis in each group.  Long-term effect of diet on immobility duration not assessed. |

NMDA: N-methyl D-aspartate

TREM-2: Triggering receptor expressed on myeloid cells-2

***Supplementary Table 2:*** *Clinical studies investigating ketogenic diets in severe mental illness*

| **Lead author (Year)** | **Study design** | **Population** | **Intervention** | **Duration** | **Minimum ketone monitoring frequency per week** | **Key findings** | **Limitations** |
| --- | --- | --- | --- | --- | --- | --- | --- |
| **Schizophrenia and bipolar disorder** | | | | | | | |
| Sethi et al, 2024 [40] | Uncontrolled pilot trial | 23 participants recruited (21 completed), 18-75 years, 61.9% female, with a DSM V diagnosis of either schizophrenia or bipolar disorder | Standard psychiatric care  One hour of teaching on how to follow the ketogenic diet (composition by weight: 60% fat; 10% carbohydrates; 30% protein), a personal coach to answer food-related questions, and a ketogenic diet cook book | 4 months | Once weekly | 20 of 21 study completers had ketone levels above 0.5mM at ≥ 60% of measurement time points (at least once a week)  Significant improvements in mean CGI and MANSA scores across the cohort. Significant improvement in mean PHQ-9 among adherent^a^ and semi-adherent^b^ participants, but not the cohort overall.  Significant reduction in Brief Psychiatric Rating Scale scores in patients with schizophrenia (p<0.02)  Significant reduction in various metabolic health measures including mean body weight, systolic blood pressure, waist circumference, HbA1c, triglycerides and homeostatic model for assessment of insulin resistance scores across the cohort. | Small overall sample size and small schizophrenia population.  Lack of controls  Limited regulation of dietary intake  Ketone levels not checked daily and may not provide accurate measure of day-to-day adherence.  Study design does not control placebo effect  Potential confounding effect of weight loss not controlled |
| Pacheco et al, 1965 [41] | Uncontrolled pilot trial | 10 female patients, 19-63 years with treatment-resistant schizophrenia | Ketogenic diet (composition not reported) + ongoing psychiatric care (antipsychotics and electroconvulsive therapy) | 2 weeks | Not measured | Significant improvement in Beckomberga Rating Scale S-factor mean scores | Small sample size.  Ketone levels not reported.  Lack of controls, hence raters were not blinded. |
| **Bipolar disorder** | | | | | | | |
| Campbell et al, 2025 [38] | Uncontrolled pilot trial | 27 participants recruited (20 completed), aged 18-70 years, 69% female, with DSM-IV diagnosis of bipolar disorder who had been euthymic for ≥ 3 months at baseline | Standard psychiatric care + modified ketogenic diet (60-75% calories from fat; 5-7% from carbohydrates; remainder from protein)  Weekly remote dietitian consultation with individualised diet plans.  Multivitamin and calcium supplementation | 6-8 weeks | Daily | Significant positive correlations between ketone levels and mood (r = 0.21, p<0.001) and energy (r = 0.19, p<0.001). Significant negative correlations between ketone levels and impulsivity (r = -0.30, p<0.001) and anxiety (r = -0.19, p<0.001)  Significant weight loss (-4.2kg, p<0.001), BMI reduction (-1.5kg/m^2^, p<0.001) and systolic blood pressure reduction (-7.4mmHg, p<0.041).  No significant changes in Diastolic blood pressure, fasting insulin, fasting glucose, HbA1c, HDL, LDL, total cholesterol, triglycerides, CRP  Significant reductions in Glx (glutamate + glutamine) in the anterior and posterior cingulate cortices | Results may not be generalisable to non-euthymic patients with bipolar disorder.  Study design prone to expectation bias |
| Needham, 2023 [39] | Same as above (feasibility study) | Same as above | Same as above | Same as above | Same as above | 77% completion rate; All participants who completed the study (n=20) had ketosis (0.5-1 mM) within 1-7 days.  1 serious adverse event: euglycaemic ketoacidosis in a participant with type 2 diabetes on an SGLT2 inhibitor | Results may not be generalisable to non-euthymic patients with bipolar disorder. |
| **Depression** | | | | | | | |
| Gao, 2026  [42] | Randomised, placebo-controlled clinical trial | 88 participants, 18-65 years (mean age: 42.1 ± 13.1 years), 69% female, with moderately severe (PHQ-9 score ≥ 15) treatment-resistant depression | Intervention group: 3 prepared ketogenic diet meals per day (4:1 ratio of fat: carbohydrates + protein; < 30g of carbohydrates per day and 15-20% of energy from protein)  Control group: phytochemical diet (one differently coloured fruit or vegetable added each day and saturated animal fats replaced by unsaturated plant oils)  Both groups: weekly 30-minute sessions with trained staff to support adherence.  Individualised energy targets and snack portions adjusted as needed to maintain weight | 6 weeks | Twice weekly | Non-significant (p=0.05) between-group difference in mean PHQ-9 score improvement in ketogenic diet group vs control group at week 6.  No significant difference in PHQ-9 improvements between groups at week 12 (p=0.10).  Significantly greater improvement in PHQ-9 scores in participants with severe depression (PHQ-9 score , 20-27) vs participants with moderate depression (PHQ-9 score, 15-19) at week 6 (p=0.02 for difference in treatment effects).  48% of participants in the ketogenic diet group had discontinued the diet within 6 weeks of support ending. | Effect size at 6 weeks was not statistically significant and fell below study’s prespecified clinically meaningful threshold.  Ketone concentrations did not correlate with symptom improvement  Study was designed to maximise adherence within intervention period and thus provides limited information on adherence in context of routine clinical practice |
| Brietzke, 2026  [44] | Uncontrolled clinical feasibility trial | 11 female participants enrolled (8 completed, 41.4 ± 10.6 years) with moderate-to-severe depression (MADRS score ≥ 20)  7 with MDD, 1 with bipolar depression among completers | Ketogenic diet supervised by a registered dietitian  1^st^ week ketogenic diet composition by weight: 50-70% fat; 30-40% carbohydrates; protein (percentage not specified)  2^nd^ week: 71-85% fat; 5-20% carbohydrates  3^rd^ – 14^th^ week: diets adjusted based on individual preferences, financial constraints and cultural considerations  Weekly dietitian consultations to support adherence | 14 weeks (2-week induction phase; 12-week maintenance phase) | Daily | 72.7% study completion rate.  Mean blood ketone levels of 0.7-2mmol/L  Significant reduction in mean MADRS scores over the intervention (~23-point reduction from baseline; p<0.001), with 87.5% of completers reaching remission.  Side effects confined to first 4 weeks of intervention.  Gastrointestinal side effects most common (64%) including nausea, diarrhoea, constipation and stomach ache.  No serious adverse events reported. | Study completers were 8 of 89 initially contacted and are likely to be more motivated than average.  Small sample size and uncontrolled design precluding efficacy conclusions  All participants were female so results might not be generalisable across sexes  Lack of blinding raises susceptibility to expectancy effects.  Possible confounding effect of concurrent psychiatric medication |
| Decker, 2025 [43] | Uncontrolled clinical trial | 24 university students recruited (16 completed) undergoing treatment for major depressive disorder; 18-30 years (mean age: 24±3); 62.5% female | <50g/day of carbohydrate, 1.5g/kg of protein, fat comprising the rest of the calories.  Ten pre-packaged ketogenic meals at baseline.  Broth/LMNT electrolyte packs | 10-12 weeks depending on participant availability | Daily | Capillary beta-hydroxybutyrate >0.5mM in 73% of daily ketone measures  Significant improvements in mean self-reported PHQ-9 scores at week 2 (37%, p=0.013) and PHQ-9 (69%, p<0.001) and  observer-rated HRSD (71%, p<0.001) scores at weeks 10-12 from baseline.  Ketone levels did not significantly correlate with the degree of depressive symptom improvement | Single-arm design without a control or comparator diet, so improvements cannot be separated from concurrent counselling/medication, medication changes, or the passage of time.  Lack of blinding raises susceptibility to expectancy effects.  Attrition was higher than in comparable ketogenic diet trials (33%, with 8 of 24 baseline participants not completing)  Small sample size and unique study population may limit the study’s generalisability. |

^a^Adherence: blood ketone levels between 0.5 and 5 mM at ≥ 80% of measurement time points

^b^Semi-adherence: blood ketone levels between 0.5 and 5mM at 50-79% of measurement time points

DSM: Diagnostic and Statistical Manual of Mental Disorders

PHQ-9: Patient Health Questionnaire-9

CGI: Clinical Global Impression

MANSA: Manchester Short Assessment of Quality of Life

MADRS: Montgomery-Åsberg Depression Rating Scale

SGLT2: Sodium-glucose transporter-2

HRSD: Hamilton Rating Scale for Depression

HbA1c: Glycated haemoglobin
